# Effectiveness of the Pasifika Women’s Diabetes Wellness Program (PWDWP): Protocol for a Pilot Intervention and Feasibility Randomized Controlled Trial

**DOI:** 10.2196/55435

**Published:** 2024-03-11

**Authors:** Heena Akbar, Madison Contor, Winnie Niumata, Debra Anderson, Danielle Gallegos

**Affiliations:** 1 School of Public Health Faculty of Medicine The University of Queensland Brisbane Australia; 2 Centre for Childhood Research, School of Exercise and Nutrition Sciences Faculty of Health Queensland University of Technology Brisbane Australia; 3 Faculty of Medicine The University of Queensland Brisbane, Queensland Australia; 4 Logan Central Community Health Centre Metro South Health, Logan Central Queensland Health Brisbane Australia; 5 Faculty of Health University of Technology Sydney NSW Australia

**Keywords:** type 2 diabetes, Māori and Pasifika women, diabetes self-management, culturally co-design intervention, Pasifika diaspora, talanoa

## Abstract

**Background:**

Type 2 diabetes poses public health challenges for Māori and Pasifika communities in Australia. The women of these communities face a greater burden from type 2 diabetes–related mortality and comorbidities. Lifestyle modification behaviors through previous women’s wellness programs have been shown to reduce the risk of developing complications in established type 2 diabetes. The Pasifika Women’s Diabetes Wellness Program (PWDWP) pilot study, co-designed with Māori and Pasifika communities, was aimed at addressing late hospital presentations from diabetes-related complications.

**Objective:**

This study (1) examines the efficacy of women with type 2 diabetes in the intervention group for improved glycated hemoglobin (HbA_1c_) clinical levels and diabetes self-management compared with the control group from baseline (T_0_) to week 12 (T_1_) and week 24 (T_2_; postintervention) and (2) assesses the cultural adaptability, acceptability, and feasibility of the pilot intervention for future studies.

**Methods:**

This study uses a quasiexperimental design that involves a 24-week intervention. We recruited 50 Māori and Pasifika women with type 2 diabetes (25 in the intervention group from the south side of Brisbane and 25 in the control group from the north side of Brisbane) using participatory talanoa methodologies. The intervention group participated in face-to-face and virtual whānau education workshops (5 weeks) and had access to individual coaching and virtual support delivered by trained Māori and Pasifika health professionals and community health workers. The control group received usual care with their identified health provider. Both groups received copies of the PWDWP journal, fact sheets, and a health check passbook with tailored motivational text messages. An advisory committee was set up to oversee the program implementation, including protocols of engagement, health checks, and data collection in community settings. The quantitative data were collected at T_0,_ T_1_, and T_2_ with HbA_1c_ as the primary outcome measure. Secondary outcomes measured changes in diabetes self-care and body composition (eg, BMI, waist circumference). Qualitative data will ascertain the program’s feasibility and cultural adaptability using talanoa focus groups.

**Results:**

This pilot study was approved by the Queensland University of Technology Human Ethic Research Committee (5609) and began in January 2023 after participant recruitment between July 2022 and December 2022. The final data collection including the health check, focus group, and survey data was completed in November 2023, and data analysis and reporting are expected to conclude in 2024.

**Conclusions:**

This study provides a blueprint for PWDWP. Collaborative partnerships with community organizations and stakeholders are crucial for program success and suggest a potential model for targeting diabetes management for Māori and Pasifika communities, emphasizing the need for culturally relevant interventions. The findings will have significant implications for policymakers and practitioners when developing and implementing public health initiatives, particularly for communities with unique cultural nuances.

**Trial Registration:**

Australian New Zealand Clinical Trials Registry ACTRN12622001100785p; https://anzctr.org.au/Trial/Registration/TrialReview.aspx?id=384470&isReview=true

**International Registered Report Identifier (IRRID):**

DERR1-10.2196/55435

## Introduction

### Background

The global prevalence of diabetes and its threat to public health continue to increase each year. Almost 90% of the estimated 537 million people currently living with diabetes worldwide have type 2 diabetes, despite it being largely preventable [1]. The Western Pacific region accounts for over one-third of the worldwide burden of diabetes, with 6 Pacific Island Countries and Territories (PICTs) among the top 10 globally with the highest prevalence of type 2 diabetes [1,2]. Additionally, PICTs face a higher prevalence of complications from debilitating diabetes as well as a higher prevalence of undiagnosed diabetes compared with other regions [2-5]. There are disparities in diabetes and diabetes-related conditions between people from PICTs and people from high-income countries, with similar disparities in Australia between Indigenous and non-Indigenous Australians [6]. As the prevalence of diabetes is positively associated with social and economic costs to individuals, families, and society, the severity and burden of type 2 diabetes are clearly highlighted [7-10].

Type 2 diabetes remains a major public health challenge for Māori and Pasifika peoples living in Queensland, which has the largest Pasifika diaspora after New Zealand [11]. Pasifika is a collective term used for Pacific peoples in Australia. Significant disparities in diabetes prevalence exist in these populations, who are 2 to 4 times more likely to be hospitalized and die from diabetes-related complications than non-Māori and Pasifika populations [12]. Evidence indicates that culturally and linguistically diverse women, in particular, face a greater burden of type 2 diabetes–related mortality and comorbidities including cardiovascular disease, kidney disease, vision impairment, and depression [12-15]. Diabetes care and support programs are less likely to be used by Māori and Pasifika women with diabetes due to low levels of health literacy, language barriers, and a strong cultural reluctance to seek help. Lack of culturally appropriate health and social services have resulted in an avoidance of engagement by Māori and Pasifika women [15]. Māori and Pasifika women carry significant cultural responsibility including expectations of acting as carers, leaders, and nurturers within their communities, often at the expense of their own chronic disease management [15]. This has significant implications for providing accessible, low-cost, and culturally appropriate diabetes care for Māori and Pasifika women with type 2 diabetes [15].

**Figure 1:**
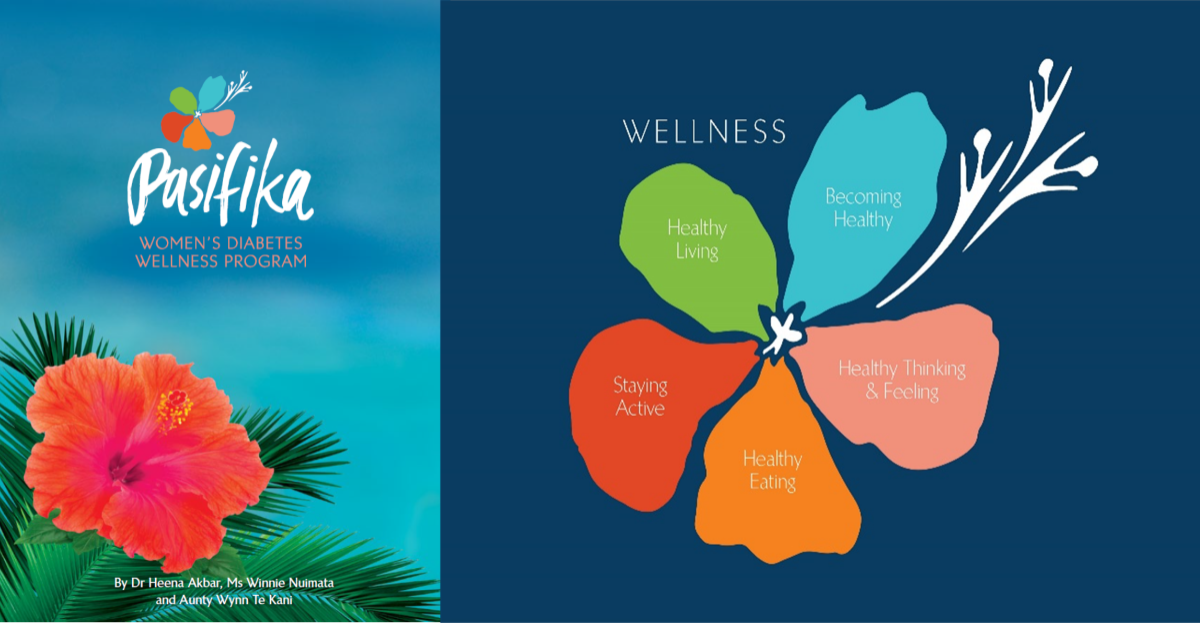
The Pasifika Women’s Diabetes Wellness Program and the "wellness" components.

Encouraging healthier behaviors in Māori and Pasifika women with type 2 diabetes could potentially improve the health and well-being of women [15,16]. Modification of personal behaviors through women’s wellness programs aimed at early intervention have been shown to reduce the risk of developing complications in established type 2 diabetes and may reduce hospitalization rates due to preventable complications [14]. A qualitative study of a multimodal behavioral intervention, the Women’s Wellness with Type 2 Diabetes, found it to be effective at supporting women with developing strategies to improve their well-being and avoid complications associated with diabetes [16]. Another study with Māori and Pasifika women with type 2 diabetes in Queensland reported effective strategies to improve self-management through culturally appropriate interventions and educational resources [15,17]. Thus, the first culturally framed intervention, the Pasifika Women’s Diabetes Wellness Program (PWDWP) emerged from these studies that informed the study’s principles and processes, including recruitment strategies and co-design of the delivery format into a whānau (family)-centered approach that harnesses digital technology [15,17]. The intervention was co-designed with Māori and Pasifika women living with type 2 diabetes in Queensland, underpinned by social cognitive theory and the Fonofale Pasifika Health Model that focuses on family, culture, community, and spirituality using talanoa, a Pacific form of dialogue, to promote sustainable self-management of type 2 diabetes [17-20]. This 24-week program is whānau-centered with 5 diabetes self-care behavior and wellness components (Figure 1) and delivered by trained Māori and Pasifika health professionals. The program takes into consideration the cultural shame associated with acknowledging the disease and tailors the interventions using talanoa as the key strategy to reach a shared meaning for behavioral changes [20].

The program team has previously ensured successful translation of interventions into practice through meaningful engagement with consumers, policymakers, health service managers, and clinicians [15,17,18,20-22]. This program will further consolidate collaborative partnerships between clinicians, health service providers, Māori and Pasifika communities and organizations, and consumer advocates. The study will evaluate the clinical benefits of the intervention and will target improvements in glycated hemoglobin (HbA_1c_; measured using a point-of-care device [18]) and diabetes self-care including diet, physical activity, stress and medication management, and routine health checks of Māori and Pasifika women with type 2 diabetes from data collected via validated questionnaires using Qualtrics [24]. The study builds on a robust and systematic program of work, beginning with scoping studies, needs assessments, and early wellness trials with women [15-17].

### Research Aim

The pilot study will evaluate the feasibility, acceptability, delivery, and effectiveness of PWDWP using mixed methods, in partnership with Māori and Pasifika community organizations and key stakeholders in southeast Queensland.

### Objectives

The primary objective is to examine efficacy, defined as the women with type 2 diabetes in the intervention group having improved clinical HbA_1c_ levels from baseline (T_0_) to week 12 (T_1_) and week 24 (T_2_) postintervention compared with the control group.

The secondary objective is to determine whether, compared with control participants, the intervention group achieves changes to bring body composition measurements closer to the recommended healthy range for specific cultural groups (eg, BMI ≤30 kg/m^2^ and waist circumference <80 cm based on World Health Organization criteria) and improved diabetes self-care scores on diet, physical activity, routine health checks, and medication adherence assessed using the validated Summary of Diabetes Self-Care Activities scale [23,25-33].

In addition, we will assess the potential for success of the proposed intervention by examining accessibility, acceptability, uptake, and cultural adaptability of the intervention; sustainability of and adherence to the intervention over time; participants’ perceptions of measurement burden; the effectiveness of the planned recruitment strategy and retention of participants; and documentation of women’s experience with PWDWP through digital stories.

### Hypotheses

H1 is that, compared with control participants, intervention participants who receive intensive whānau face-to-face and virtual support for type 2 diabetes management will have improved HbA_1c_ levels at 12 weeks and 24 weeks (postintervention sustainability).

H2 is that, compared with control participants, intervention participants who receive intensive whānau face-to-face and virtual support for type 2 diabetes management will have reduced waist circumference and BMI at 12 weeks and 24 weeks.

H3 is that, compared with control participants, intervention participants who receive intensive whānau face-to-face and virtual support for type 2 diabetes management will have improved diabetes self-care at 12 weeks and 24 weeks.

Data collection occurred at 3 time points: T_0_ (before the commencement of the pilot program, week 1), T_1_ (postintervention, week 12), and T_2_ (postintervention, week 24). Data on the feasibility, accessibility, and affordability of the PWDWP were collected via satisfaction surveys for the intervention group at the end of the workshop (week 5), week 12, and week 24. For the control group, satisfaction surveys were completed in week 12 and week 24.

Qualitative data obtained through talanoa focus groups with participants will determine measurement burden, acceptability, potential translation, and ease of intervention access. Feasibility covers how the participants were referred to the program, time taken to complete questionnaires, any strengths or weaknesses of the intervention, its cultural relevance, experience with accessing the intervention, perceptions of the digitally delivered coaching sessions and online virtual support sessions as well as the face-to-face intervention, and any suggested improvements to the study design or intervention.

## Methods

### Advisory Committee

An advisory committee (AC) was established to oversee the pilot study, including its setup, ongoing management, promotion, implementation, and evaluation with outcome deliverables including appropriate dissemination. The AC includes members of the Māori and Pasifika communities including the Pasifika Women’s Alliance and Pacific Island Council of Queensland and key organizations such as the Good Start Program for Māori and Pacific Islander Peoples (Children’s Health Queensland) and academia (eg, Queensland University of Technology [QUT] and University of Technology Sydney through the Women’s Wellness Research Program). A series of group talanoa undertaken with AC community members ensures that the pilot study is implemented and evaluated within the talanoa cultural framework. The AC meets regularly every 4 months to review the study protocols including recruitment strategies, health checks, and data collection methods and approaches and how these are disseminated. The AC provides input on the trial progress, adherence to the protocol, participant safety, and consideration of new information as well as advice on the location and place of delivery (intervention and data collection) considered to be culturally acceptable to the participants.

### Participatory Talanoa Methodologies

The research uses participatory action research using co-design and talanoa methods as the theoretical and cultural framework in the collection and analysis of data.

Talanoa is a way of communicating in a Pacific context. It has been described as the co-construction of knowledge through respectful, reciprocal conversation. It is a process enabling critical discussions and knowledge construction allowing for rich contextual and inter-related information to surface as co-constructed stories [19]. Vaioleti [19] uses the following metaphor of kakala:

- “Toli” is deciding on, selecting, and picking the flowers, equating to recognizing a problem, how participants are chosen, and which data are collected and analyzed [19].
- “Tui” is the process of making and weaving the kakala, in which the stories and emotions of deep talanoa encounters are integrated and synthesized. This involves deciding on the type and amount of data to use, how the data are arranged in relation to each other, and how they are presented.
- “Luva” is the giving away of the kakala, when the research is given for the benefit of the community, where solutions can be found.

In this context, community engagement, including recruitment, data collection, and analysis processes, is a form of talanoa. All data are analyzed through talanoa with the AC and community researchers. In this context, talanoa is a method embedded within a participatory methodology [19] that informs the protocol of this study.

### Study Design

This pilot study is a quasiexperimental design with 2 arms: an intervention (delivered in person and online for those who preferred virtual delivery) and an attention control arm (receiving written information and text messages). The study is being conducted to trial a 12-week intervention followed by a 12-week follow-up period (over 24 weeks) delivered by trained Māori and Pasifika health professionals (diabetes educator, clinicians, community health workers [CHWs], and community researchers). The study is conducted in southeast Queensland, Australia, with Māori and Pasifika women living with type 2 diabetes who were recruited through participatory community engagement with the Māori and Pasifika community leaders and organizations.

**Figure 2:**
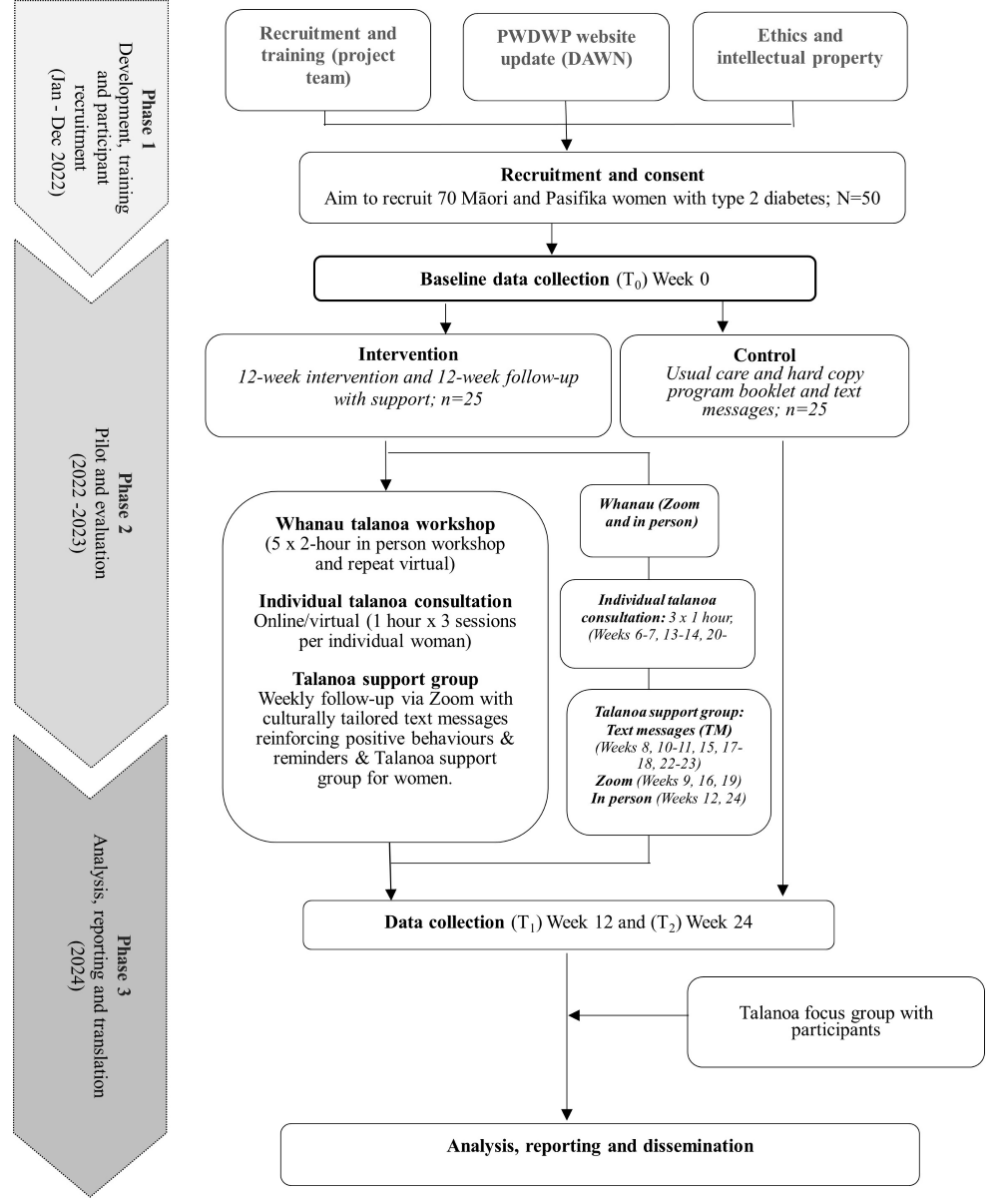
Overview of the Pasifika Women’s Diabetes Wellness Program (PWDWP) pilot study in southeast Queensland, Australia (2022-2024).

The study uses participatory talanoa methodologies using in-depth interviews and surveys and involves an in-person talanoa education workshop (5 weeks) as well as virtual workshops (5 weeks for those participants not in Brisbane and unable to attend in person), 3 online individual talanoa consultations (coaching) via Zoom, and an online virtual talanoa support group with motivational SMS text messages. The control participants received the usual care with their identified health professionals, a hard copy of the program materials (which includes a program journal, recipe book, fact sheets, and passbook), as well as general motivational SMS text messages. The process evaluation includes satisfaction surveys after the workshop sessions at week 5, week 12, and week 24 as well as a talanoa focus group with the participants after the intervention. Figure 2 is an overview of the PWDWP pilot study in southeast Queensland, Australia (2022-2024). The detailed intervention is described in the following sections.

### Intervention

The PWDWP intervention includes a whānau talanoa workshop, individual talanoa consultations, and a talanoa support group and text messages.

#### Whānau Talanoa Workshop

Five 2-hour sessions covered the wellness components. Whānau talanoa workshops provided interactive knowledge-based education on targeted health knowledge and behaviors with culturally specific tools to manage type 2 diabetes within family, community, and spiritual contexts (see Table 1).

**Table 1:** Whānau talanoa education workshops on diabetes wellness components delivered by a trained Pasifika diabetes educator and community health workers.

Education component	Content delivery	Weeks delivered
Becoming Healthy	This workshop provides practical information on type 2 diabetes and its symptoms, risk factors, diagnosis, treatment and management, medication, and self-care. This session also covers practical and cultural ways to deal with issues related to the shame and stigma of developing type 2 diabetes and the impact of denial.	1
Healthy Thinking & Feeling	This workshop provides information and tools to deal and cope with stress and barriers and ways to improve wellness that incorporate elements of Pasifika values, spirituality, and faith.	2
Healthy Eating	This workshop provides information and tools for healthy eating with a self-care healthy eating plan and strategies for managing cultural hospitality requirements without losing face.	3
Staying Active	This workshop covers ways to get moving, types of exercises to do with your family, and practical tips to remain motivated to stay active while managing family and community obligations.	4
Healthy Living	This workshop encourages, motivates, and maintains healthy living including looking after your bones, eyes, kidney, and heart; sleep; and menopause with spirituality, family, and community well-being.	5

#### Individual Talanoa Consultations

Three 1-hour, 1-on-1 sessions were conducted. The first session consisted of a routine check-up, appropriate screenings, and a discussion to facilitate tailored education, goal setting, and specialist advice to support individualized plans and goals for a healthy weight, diet, physical activity, stress management, sleep, and medications. The second session revisited goals set during session 1 and, if necessary, discusses prevention strategies and identification of barriers. The third session reviewed participant progress including barriers to self-management, reinforcement of positive behaviors, and setting future goals for maintenance.

#### Talanoa Support Group and Text Messages

A virtual peer support group facilitated by CHWs encouraged and supported women with targeted topics including stress management, barriers, understanding family situations, mindfulness, nutrition and diet, importance of health checks, regular visits to general practitioners and specialists, monitoring blood glucose levels, medication adherence, and maintenance. Motivational SMS text messages were sent regularly to reinforce key messages.

### Cross-Intervention and Control Conditions

The intervention group received the complete intervention over the 24 weeks and had access to an interactive program journal (hard copy and electronic), a recipe book, a passbook for health check, and fact sheets as well as text messages with reinforcing positive motivational behaviors (Table 2). The participants also had access to a website that includes culturally developed resources (such as podcasts, digital stories, and information materials).

**Table 2:** The intervention component for the Māori and Pasifika women with type 2 diabetes from the south side of Brisbane, Queensland, Australia.

Intervention components	Weeks (1-24)
Whānau talanoa workshops (in person and virtual)	1-5^a^
Individual talanoa consultation (coaching via Zoom)	6-7, 13-14, 20-21
Motivational SMS text messaging	8, 10-11, 15, 17-18, 22-23
Talanoa support group (via Zoom)	9, 16, 19
Data collection (T_0_, T_1_, T_2_; in person)	0 (baseline), 12, 24
Talanoa focus group (in person)	24

^a^Offered either (1) in person (face to face) with women or (2) online (virtual) for those who cannot attend in person.

**Figure 3:**
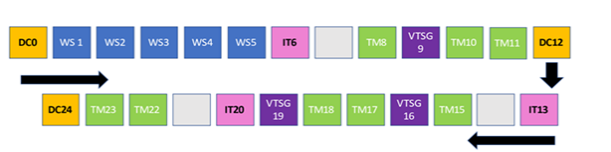
Intervention participant journey of Māori and Pasifika women with type 2 diabetes from the south side of Brisbane, Queensland, over 24 weeks (January 2023 to November 2023), including five 2-hour education workshops (WS; either face to face or virtual), 3 individual talanoa (IT) consultations, and 3 virtual talanoa support group (VTSG) sessions with text messages (TM). DC: data collection.

The program provided all necessary health promotion content and supported participants with logging relevant health information in the journal (either hard copy or an electronic version). A weekly diet, physical activity, and diabetes self-care checklist encouraged participants to plan ahead. The interactive website aims to reinforce educational content, enabling home monitoring of measurable health indicators, and allowed participants to fill out questionnaires and other data. The website will also be adapted for all computing platforms, including mobile phones for future studies. Figure 3 illustrates the intervention participant journey of Māori and Pasifika women with type 2 diabetes from the south side of Brisbane over 24 weeks (January 2023 to November 2023).

**Figure 4:**
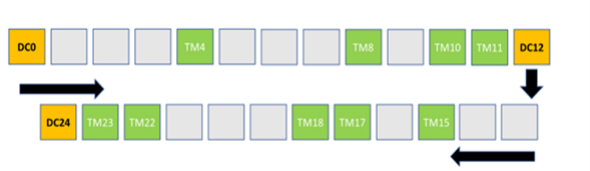
Control participant journey of Māori and Pasifika women with type 2 diabetes from the north side of Brisbane over 24 weeks (January 2023 to November 2023), including usual care with their preferred health provider and motivation text messages (TM). DC: data collection.

The control group received their usual care with the hard copy of the program materials (ie, program journal, recipe book, fact sheets, and passbook) and SMS text messages (Table 3). At the completion of the intervention, control participants received a modified 1-day workshop covering talanoa education workshops. Figure 4 illustrates the control participant journey of the Māori and Pasifika women with type 2 diabetes from the north side of Brisbane over 24 weeks (January 2023 to November 2023).

**Table 3:** The control component for the Māori and Pasifika women with type 2 diabetes from the north side of Brisbane, Queensland, Australia.

Control components	Weeks (1-24)
Usual care with a hard copy program booklet (participants will be asked to document in the study journal [encouraged daily or weekly] but will be at their own discretion)	1-24
Motivational SMS text messaging	4, 8, 10-11, 15, 17-18, 22-23
Data collection (T_0_, T_1_, T_2_; in person)	0 (baseline), 12, 24
Talanoa focus group (in person)	24

### Inclusion and Exclusion Criteria

Inclusion criteria included Māori and Pasifika women with type 2 diabetes, aged ≥18 years, diagnosed with type 2 diabetes, and with access to the internet. Participants were excluded if they were pregnant and diagnosed with gestational diabetes, had a high-risk pregnancy, or were not diagnosed with type 2 diabetes.

### Locations and Online Forums

#### Whānau Talanoa Intervention (Onsite)

The onsite face-to-face whānau talanoa sessions occurred in the community setting as recommended by the AC to ensure they were culturally acceptable to the participants in a whānau setting that is inclusive of the community.

#### Online Virtual Talanoa Intervention

The online sessions occurred via the Zoom platform over 5 weeks and covered the same content delivered in the face-to-face session. The Zoom setup was organized and managed through the secure QUT system, which enabled the project coordinator to host and manage each session and recordings. All the recordings and transcripts are stored with password protection and security in the QUT shared drive project folder, which can be accessed by the project team only (principal investigator and coordinator).

#### Individual Talanoa Consultation

The individual talanoa consultations (either via Zoom or telephone) were delivered by a trained CHW educator and managed through case notes that were uploaded (including Zoom recordings) to a secure project folder within the QUT shared drive.

### Study Population and Setting

#### Study Type

This is a participatory community-based study. Māori and Pasifika women living with type 2 diabetes were recruited from the Pasifika communities in southeast Queensland through a range of organizations, representing various sectors of the Māori and Pasifika communities in Queensland. The intervention and control groups were geographically distinct to minimize cross-communication, as members within the same community socialize, and this is the design the AC recommended as culturally appropriate.

#### Intervention Group

The intervention participants were recruited from the south side of Brisbane, including Logan, Ipswich, and Gold Coast, and geographically separated from the control group. Inclusion criteria for intervention participants were women aged ≥18 years from the south side of Brisbane, diagnosed with type 2 diabetes, and with access to the internet.

#### Control Group

The control participants were recruited from the north side of Brisbane, including the Sunshine Coast. Inclusion criteria for control participants were women aged ≥18 years from the north side of Brisbane who have been diagnosed with type 2 diabetes.

The exclusion criteria for intervention and control groups were pregnant women diagnosed with gestational diabetes or with a high risk of pregnancy. Women from each arm were also excluded depending on the location (eg, South side women were excluded from the control group and vice versa).

#### Sample Size

The sample size was calculated based on previous studies for detecting a difference of 1% reduction in HbA_1c_ (primary outcome) with a standard deviation of 1.4% in the intervention group. A significance level (α) of .05 was considered with a study power of 80% [34]. Based on this, a sample of 64 participants was needed (32 for the control group and 32 for the intervention group), with a target sample of 90 participants to compensate for a 40% attrition rate. Although the program received approximately 90 expression-of-interest responses, challenges with community engagement, complexities arising from COVID-19, and participant dropout resulted in a total of 50 participants, with 25 in the control group and 25 in the intervention group who enrolled and completed the pilot program.

#### Community Settings

The program for the intervention group was provided in community settings: Pasifika Te Haus in Runcorn and Adam Gould Community Centre in Logan on the south side of Brisbane. There is a large Māori and Pasifika population in southeast Queensland. For the control group, the first health checks and data collection occurred at a Pasifika Community Church, but for the follow-up visits, home visits were undertaken as requested by the participants. The community locations were recommended by the AC.

### Recruitment Strategies

Recruitment for both intervention and control groups was multimodal and through distribution of study flyers, email invitations, and advertisements via social media (Facebook, websites); local ethnic radio stations; newsletters; and community networks including church groups, organizations, and Māori and Pasifika health workers. Recruitment also occurred through presentations and dialogue with community partners and organizations, including the Pasifika Women’s Alliance Inc, Oceania Pacific Health Association, and Multicultural Communities Council Gold Coast, who have representatives on the AC, and they promoted the study through their networks and social media.

### Ethical Considerations

The PWDWP was approved by the QUT Human Ethic Research Committee (5609). All data will be aggregated, with no individuals able to be identified. Collected data will be coded for analysis. The anonymity and privacy of all participants will be maintained through the use of fictional names, unique participant identification numbers, and secure data storage. All data will be stored securely on a password-protected research drive as per QUT’s management of research data policy and the National Health and Medical Research Council guidelines for trials, with access restricted to only the principal investigator and co-investigator.

Potential participants were contacted to discuss the study and consent processes. Those who agreed received a “participant information pack” with an introduction letter detailing the program information, timelines, demographic questionnaires, and participant consent information. The project officer conducted 2 follow-up phone calls with each participant before the intervention to finalize details regarding the program; confirm the mode of preference for participating in part 1 (in person or online) of the intervention; and discuss issues or concerns with participation, access to their own GP, and dropout as well as any other issues that the women would like to discuss. Participants who consented to participate were asked to get a medical checkup prior to the start of the program and raise any concerns with the project officer. All participants (intervention and control) who consented were allocated a unique ID number and registered in a trial database. Any reasons for nonparticipation were recorded. The original informed consent for the primary data collection allows secondary analysis without additional consent.

Any cultural, spiritual, and personal concerns about disclosure in relation to cultural and religious beliefs especially around stigma associated with a diabetes diagnosis were discussed and clarified with participants before obtaining consent to disclose or use any sensitive information. Cultural sensitivity and respect form the underpinning guiding value in this research process especially when working with Māori or Pasifika families from various ethnic and religious backgrounds. This also included talanoa style of conversations to clearly explain the reason for choosing the 2 different locations (sites) for recruitment of control and intervention participants and the benefits and risks with each group allocation.

Although we acknowledge that the Pasifika communities are well connected and share familial relationships, there was a possibility for the lack of confidentiality. One way this is addressed is by discussing with participants the importance of anonymity while at the same time acknowledging that this can be difficult in close-connected collective communities. This is a limitation to the study.

All participants, including control and intervention participants, were remunerated ("Koha") with gift vouchers (A$20 [US$13.05] for the health check and A$30 [US$19.57] for the talanoa focus group) to compensate for for their out-of-pocket expenses, travel time, and costs for participating at each data collection.

### Data Collection and Analysis

#### Training of Community Health Workers, Community Researchers, and Research Team

Before the study commenced (July 2022 to December 2022), the Pasifika CHWs, health professionals, facilitators, and project team (including community researchers) received training on cultural protocols for engagement, community values, working with families across different Māori and Pasifika communities including the use of talanoa methods and approaches to coaching and delivering interventions within cultural Pasifika health model frameworks, and the protocols to be followed. 

Training was undertaken by the principal investigator and diabetes educator in the form of role play, storytelling, and interactive activities using scenarios. The AC was invited to participate in the training as well as to contribute and share cultural experiences and advice in relation to working with Māori and Pasifika communities in a culturally safe and sensitive manner. All training was recorded, minutes were taken, and registration attendance was noted. The community researchers were also provided a step-by-step instruction manual on data collection.

#### Quantitative Outcomes

Quantitative data were collected through face-to-face or in-person sessions using online questionnaires at 3 time intervals (T_0_, T_1_, T_2_) between January 2023 and December 2023 in a community-based location for both intervention and control participants. Data collected included anthropometric (height, weight, waist circumference, BMI), sociodemographic (age, marital status, employment status, income, post code, ethnicity), diabetes (self-care, self-efficacy), physical activity and dietary changes, and clinical (HbA_1c_ using the DVA Vantage point-of-care HbA_1c_ kit, Siemens) data [23,25-28, 31-33]. The diabetes educator and CHWs collected anthropometric and clinical data, and the community researchers completed an online Qualtrics survey [24] using a tablet. Each participant was given a unique identifier.

#### Quantitative Data Analysis

Quantitative data analysis will be conducted in 2 steps. Raw data will be examined for missing entries, inconsistencies, and data entry errors. Any identified problems will be corrected by checking the data against the hard copy questionnaire. To further ensure reliability and accuracy of the data entry, the researcher will randomly re-enter 10% of the collected data and compare the 2 files [34].

Data are analyzed using SPSS 24 (IBM Corp), and missing data, outliers, normality, and multicollinearity will be examined using procedures outlined by Pallant [34,35]. Methods of data analysis will include descriptive, bivariate, and logistic regression analyses. The levels of statistical significance will be set at P=.49 to reduce the risk of type 1 errors [34]. The analyses will identify changes in the primary and secondary outcomes from baseline to week 12 and week 24 for Māori and Pasifika women with type 2 diabetes who completed the intervention. Descriptive statistics will examine participants’ baseline measures and characteristics and will also explore trends in the data over time. Means (SDs) will be reported for continuous variables, and percentages and frequencies will be reported for categorical variables. Independent *t* tests will be used for continuous variables, and chi‐square tests for categorical variables will be used to compare mean groups. Normality of the included variables will be evaluated using the Shapiro‐Wilks normality test, and a nonparametric test (Mann‐Whitney *U* test) will be used if data are not normally distributed to detect differences between groups. A 2‐sided P=.49 will be considered significant for all analyses [34,35].

The study will also provide initial parameter estimates for the primary endpoint, from which sample size calculations can be performed to determine sufficient power for the main trial. The primary endpoint between group differences at 12 and 24 weeks will be estimated using a linear mixed model with a treatment-time interaction. Although aiming for an intention-to-treat analysis, we recognize there were withdrawal from the study. A prespecified multiple imputation model will be used to obtain intention-to-treat estimates. Sensitivity analyses will explore the impact of nonignorable missing data. Analysis of secondary endpoints will follow the same approach in which changes will be compared between intervention and control groups using linear mixed models.

#### Process Evaluation

The process evaluation includes satisfaction surveys and a talanoa focus group involving the intervention and control participants to evaluate the feasibility of the program. This includes evaluating the training of Pasifika CHWs, community researchers, and the project team; types of delivery (in person and online or virtual for education sessions); whether core messages through text messaging reinforces a positive mindset and behaviors; the talanoa approach via online and in-person delivery of the intervention; shared understanding and core messages across the project team including CHWs, community researchers, and the workshop facilitator; and satisfaction with the program assessed at T_1_ and T_2_. In addition, we will capture where the participants heard about the program, if accessibility to the program was easy, some of the challenges with the program, and what the participants would change about the program.

#### Qualitative Outcomes

Qualitative data were collected using a talanoa focus group to gather information about the participants’ experiences with the program and its acceptability, usefulness, barriers and enablers to participation, feasibility of time commitment, burden, and cultural relevance. Data gathered will explore time taken to complete data surveys, strengths and weaknesses of the intervention, perceptions of the digitally delivered sessions, and any suggested improvements to the study design or intervention as well as whether control participants had heard about the program, spoken to the intervention participants, and the influence of this on their participation.

#### Qualitative Data Analysis

Talanoa focus groups were conducted in a community setting, facilitated by community researchers and the principal researcher using a guide co-developed with the AC. The talanoa discussions, which lasted 90 minutes, were audio-recorded using a digital recorder and transcribed verbatim for analysis [20]. The trained community researcher took detailed notes using the guide. Immediately after the talanoa focus group session, the notetaker and community researcher summarized and prepared debrief notes that highlighted key points from the group. These were presented back to the focus group. The participants were also given the opportunity to read a copy of what they said and make any changes [20].

NVivo software will be used for data management and manual coding or identifying recurring keywords and phrases to ascertain measurement burden, acceptability, potential translation, and ease of intervention access. Co-analysis using inductive and deductive approaches will involve participatory talanoa processes with the Pasifika community researcher and participants iteratively [19,20]. The key themes from the co-analysis will be collated and presented to the AC for discussion. Patterns of meanings and interpretations during the analysis processes will be further deliberated by the AC to ensure community and cultural contexts are considered. Participants will be given progress feedback of the results and study outcomes. Giving feedback will be an integral part of the participatory research cycle in which the participants will have the opportunity to construct, reconstruct or retell, and reflect on the stories of their experience in the pilot program including both positive and negative feedback. A copy of the result findings and action outcomes will be disseminated to the participants, health providers, community organizations, and research team.

## Results

### Pilot Program

The pilot program began in February 2023 with 50 participants who were enrolled between January 2022 and January 2023. For the intervention group, 35 women were recruited, and there were 10 dropouts, resulting in 25 women in the intervention group. For the control group, 27 women were recruited, and there were 2 dropouts, resulting in 25 women in the control group.

Data were collected from January 2023 to December 2023.

Methods of quantitative data analysis will include descriptive, bivariate, and logistic regression analyses. The focus group was conducted in November 2023, and the results are being analyzed using an inductive-deductive approach. Film and digital story production are underway and expected to be complete in April 2024. Data analysis is planned to occur from March 2024 to July 2024.

### Importance of the Results

The study participants in the intervention and control groups are from diverse Pacific cultural backgrounds and include Fijians, Fiji Indians, Samoans, Māori, Tongans, Cook Islanders, and those who identify with more than one cultural group. The anticipated results from the quantitative and qualitative analyses would highlight the relevance that culturally co-designed programs could lead to improved diabetes self-care outcomes including clinical HbA_1c_ levels, positive changes in body composition measurements such as BMI and waist circumference, and enhanced diabetes self-care scores within the intervention group.

### Dissemination Plan

Results are expected to be published as early as December 2024 and extend through July 2025. The study findings will be disseminated through presentations at state and national meetings (eg, Queensland Women’s Health Forum, Australian Public Health), international meetings and conferences (eg, the International Diabetes Congress), and publication in peer-reviewed journals. In addition, we will work closely with Māori and Pasifika community partners to identify strategies to disseminate study findings in the community through community talanoa forums and meetings. Other social media network platforms and local community radio will be used to disseminate the study findings.

## Discussion

Type 2 diabetes is a costly, rapidly growing, and largely preventable disease. Encouraging healthier lifestyles in Māori and Pasifika women with type 2 diabetes could potentially improve the health and well-being of women, prevent complications, and reduce health expenditure.

This paper describes the protocol of a quasiexperimental pilot study based on a culturally co-designed whānau-centered intervention for Māori and Pasifika women living with type 2 diabetes in Queensland, Australia [15]. The study consists of 2 arms (intervention and control) to trial a 12-week intervention followed by a 12-week follow up-period delivered by trained Māori and Pasifika health professionals and CHWs.

This study will report the clinical benefits of the intervention and will target improvements in HbA_1c_ and diabetes self-care including diet, physical activity, medication management, and routine health checks for Māori and Pasifika women with type 2 diabetes. Community-based interventions among Australian Samoans in Sydney, Australia, showed a reduction in HbA_1c_ [36], which would be promising for our study. Studies with Māori and Pasifika have shown that using community-based approaches and culturally tailored interventions are likely to improve health behaviors [37,38]. The qualitative data from the focus groups will inform measurement burden, acceptability, potential translation, and ease of intervention access, which has not been evaluated in other community-based studies with Māori and Pasifika communities in Australia. The potential for success of the proposed intervention will be examined using accessibility, acceptability, uptake, sustainability, adherence, participants’ perceptions of measurement burden, effectiveness of the recruitment strategy and retention, and the participants’ overall experience, based on women’s wellness resulting from type 2 diabetes programs [16]. This will inform the program’s cultural adaptability, acceptability, and feasibility for future studies.

This study recruited 50 Māori and Pasifika women with type 2 diabetes through community-led participatory approaches and with the input from the AC to ensure that the protocols of engagement with the community, recruitment of participants, and implementation and evaluation of the program were community-driven and culturally appropriate [20-22] Interventions that are specifically co-designed with communities ensure cultural relevance, effectiveness, and sustainability for addressing chronic conditions such as type 2 diabetes [22,39]. Equally important is the community-led participatory approach that embeds Māori and Pasifika cultural values within program design and implementation, which is pivotal to engaging and empowering communities to successfully improve health behaviors, particularly when tackling type 2 diabetes [21,22,39]. Developing health and lifestyle programs within communities and partnering with stakeholders including health services, governmental and nongovernmental organizations, and academic institutions have been shown to be essential to empowering underserved populations, particularly as the programs aim to understand and support behavioral changes leading to better health and well-being outcomes [21,22,38,40]. This program supports the participants in developing knowledge and practical skills for preventing and managing type 2 diabetes as well as overcoming challenges that participants and their families may face in living a healthy life [21]. As a result, this has enabled the production of 5 digital stories of women from diverse Pasifika backgrounds in the intervention, documenting their experiences and journeys, which will be used as a health promotion resource for the program in future studies.

Despite these strengths, this pilot study has limitations. One inherent limitation is the potential for cross-communication. Due to the close nature of the included communities, it is likely that they will speak to each other regarding the program. For this reason, the intervention and control groups were allocated based on geographic location to attempt to minimize this. The sample size is too small to assess the extent to which the metabolic improvements are due to self-management (eg, lifestyle change) or clinical management and may also result in nonsignificant *(P*<.05) results for some of the measurement outcomes. In addition, community-based studies are difficult to conduct in a rigorous randomized controlled trial (RCT) design without significant funding, for which pilot data are important to inform study design, including power calculations [36]. However, this study was undertaken as a pilot study to provide a proof of concept prior to undertaking a stepped-wedge RCT or multicenter RCT. Regardless of this, the outcomes of this pilot study will provide valuable information regarding culturally appropriate implementation and evaluation. The lessons learned in this pilot trial will help us to plan the larger trial with regard to recruitment strategies, intervention implementation, and data collection. The evidence-based synthesis of findings with community participants and researchers, including dissemination of the pilot study outcomes, will have future applications aimed at leading to larger studies implementing a state or nation-wide program and undertaking translation research with the potential to develop innovative digital or virtual delivery of the program with a health data collection interphase, website platform for virtual delivery, and use of a mobile app.

In conclusion, this study provides a blueprint for PWDWP and proposes a model for evaluating intervention efficacy and effectiveness in partnership with Māori and Pasifika community organizations and key stakeholders in southeast Queensland, Australia. The study findings will have significant implications for policy makers and practitioners when implementing a diabetes management program for Māori and Pasifika communities, emphasizing the need for culturally relevant interventions. Collaboration with communities and stakeholders is crucial when developing diabetes management and lifestyle programs, particularly for communities with unique cultural nuances. The findings of this study may suggest a potential model for addressing health disparities in other culturally and linguistically diverse populations.

## Appendix

Multimedia Appendix 1 Peer Reviewed RR1 Registered Report.

Multimedia Appendix 2 SPIRIT PROTOCOL Checklist.

## Abbreviations

AC: advisory committee
CHW: community researchers
HbA1c: glycated hemoglobin
PICTs: Pacific Island Countries and Territories
PWDWP: The Pasifika Women’s Diabetes Wellness Program
QUT: Queensland University of Technology
RCT: randomized controlled trial
T0: baseline
T2: week 24

## Data Availability

The data sets generated during and/or analyzed during this study are available from the corresponding author on reasonable request.
