# Correction: Effectiveness of the Pasifika Women’s Diabetes Wellness Program (PWDWP): Protocol for a Pilot Intervention and Feasibility Randomized Controlled Trial

**DOI:** 10.2196/65322

**Published:** 2024-08-26

**Authors:** Heena Akbar, Madison Contor, Winnie Niumata, Debra Anderson, Danielle Gallegos

**Affiliations:** 1 School of Public Health Faculty of Medicine The University of Queensland Brisbane Australia; 2 Centre for Childhood Research, School of Exercise and Nutrition Sciences Faculty of Health Queensland University of Technology Brisbane Australia; 3 Faculty of Medicine The University of Queensland Brisbane, Queensland Australia; 4 Logan Central Community Health Centre Metro South Health, Logan Central Queensland Health Brisbane Australia; 5 Faculty of Health University of Technology Sydney NSW Australia

In “Effectiveness of the Pasifika Women’s Diabetes Wellness Program (PWDWP): Protocol for a Pilot Intervention and Feasibility Randomized Controlled Trial” (JMIR Res Protoc 2024;13:e55435) the authors made three additions.

A section of text has been added to the “Acknowledgements” that reads as follows:

The MMAS-8 Scale (U.S. Copyright Registration No. TX0008632533), content, name, and trademarks are protected by US copyright and trademark laws. Permission for use of the scale and its coding is required. A license agreement is available from MMAR, LLC., www.moriskyscale.com

Additionally, two citations will be added after Reference 28 in the second paragraph of the “Objectives” section which appeared as:

The secondary objective is to determine whether, compared with control participants, the intervention group achieves changes to bring body composition measurements closer to the recommended healthy range for specific cultural groups (eg, BMI ≤30 kg/m^2^ and waist circumference <80 cm based on World Health Organization criteria) and improved diabetes self-care scores on diet, physical activity, routine health checks, and medication adherence assessed using the validated Summary of Diabetes Self-Care Activities scale [23,25-31].

This will now be changed to read as:

The secondary objective is to determine whether, compared with control participants, the intervention group achieves changes to bring body composition measurements closer to the recommended healthy range for specific cultural groups (eg, BMI ≤30 kg/m^2^ and waist circumference <80 cm based on World Health Organization criteria) and improved diabetes self-care scores on diet, physical activity, routine health checks, and medication adherence assessed using the validated Summary of Diabetes Self-Care Activities scale [23,25-33].

The references added are as follows, with all subsequent references reordered accordingly:

29. Berlowitz DR, Foy CG, Kazis LE, Bolin L, Conroy LB, Fitzpatrick P, et al. for the SPRINT Study Research Group. Impact of Intensive Blood Pressure Therapy on Patient-Reported Outcomes: Outcomes Results from the SPRINT Study. N Engl J Med 2017; 377:733-44.

30. Bress AP, Bellows BK, King J, Hess R, Beddhu S, Zhang Z, et al, for the SPRINT Research Group and the SPRINT Economics and Health Related Quality of Life Subcommittee. Cost- Effectiveness of Intensive versus Standard Blood Pressure Control. N Engl J Med 2017; 377:745-55.

The correction will appear in the online version of the paper on the JMIR Publications website on August 26, 2024, together with the publication of this correction notice. Because this was made after submission to PubMed, PubMed Central, and other full-text repositories, the corrected article has also been resubmitted to those repositories.

