# Supplementary material for: Effectiveness of the Pasifika Women’s Diabetes Wellness Program (PWDWP): Protocol for a Pilot Intervention and Feasibility Randomized Controlled Trial
Source: JMIR Res Protoc. 2024 Mar 11;13:e55435. doi: 10.2196/55435 (PMC10964139; doi:10.2196/55435)
Supplement: Multimedia Appendix 1 [file resprot_v13i1e55435_app1.pdf]

Diabetes Australia Research Program

Feedback for 2022 Grant Round

Grant ID: Y22G-AKBH

Project Title: Effectiveness of a Pasifika Women's Diabetes Wellness Program (PWDWP)

Diabetes Australia received a total of 273 applications for the 2022 grant round which was highly competitive and applications were of a very high standard.

The number of applications received for each grant type:
253 General Grants
6 Millennium Award -Type 1 Diabetes
14 Millennium Award – Type 2 Diabetes

Diabetes Australia provides Reviewer comments (feedback) in good faith and on an as received basis (unedited). Reviewer details and scores will not be provided and we will not enter into any further correspondence with regard to this feedback.

We thank you for your interest in our research program and hope that this information will be of assistance.

Comments

Clear objectives and good feasibility. Highly important work in a area of very high need - culturally diverse diabetes program.

Well-designed culturally relevant diabetes education program - co-designed with the intended audience and appears to include many important cultural themes and based on a prior body of scoping work.

Timeline is overly ambitious for a 6-month trial - i.e., assumes all 80 women can be recruited and complete the 3-month intervention within 3 months? Unclear that 'contamination' is sufficient justification for this to be a non-randomised trial? If rolled out nationally, would expect contamination. Powered to detect 1% reduction in HbA1c over 12 weeks - may be overly ambitious (and therefore underpowered). Proposal does state this HbA1c reduction is based on prior studies but is not referenced. If 'feasibility' is a true goal (as per Translation section), then outcomes should include feasibility metrics e.g., a feedback survey &/or focus groups, costing assessments, session attendance rates etc not just clinical metrics.

This randomised control pilot research project is examining the impact of a culturally relevant education intervention, for women of Pacfiic Islander heritage, on HbA1c, anthropometric measures and diabetes self care compared to a control group who will receive one component of the intervention and usual care.

The researchers have the track record of oral presentations at conferences but only evidence of one research grant; positively, they are identified and accepted by the Islander community. By providing this tailored program which is culturally relevant, participants will be given the best opportunity to engage. To reduce "contamination" the researchers will recruit from 2 different geographical areas. A potential bias and thus generalisibility to the whole Pacifiic Island community is that the subjects must be computer savvy and experienced. It is known that it can be difficult to engage some cultural groups in mainstream diabetes education and management.

Whether this program would be suitable to be rolled out nationally for this cultural group is unknown and untested. However if this pilot is successful then a larger research study will be developed. It has the potential to help many Islansders living with diabetes and their families in Australia. The authors have not addressed drop out, nor has the time spent with each group been addressed. As this is only a 12 week intervention it will be important for longer term evaluations in a larger trial to assess clinical outcomes overtime and their long term sustainability. The budget seems appropriate. It is a reasonalble research project that if done well will provide a platform for a larger trial.
